# *QuickStats:* Percentage* of Adults Aged ≥18 Years Who Were Advised During the Past 12 Months by a Doctor or Other Health Professional to Increase Their Amount of Physical Activity or Exercise,^†^ by Age Group and Sex — National Health Interview Survey, United States, 2022^§^

**DOI:** 10.15585/mmwr.mm7306a5

**Published:** 2024-02-15

**Figure:**
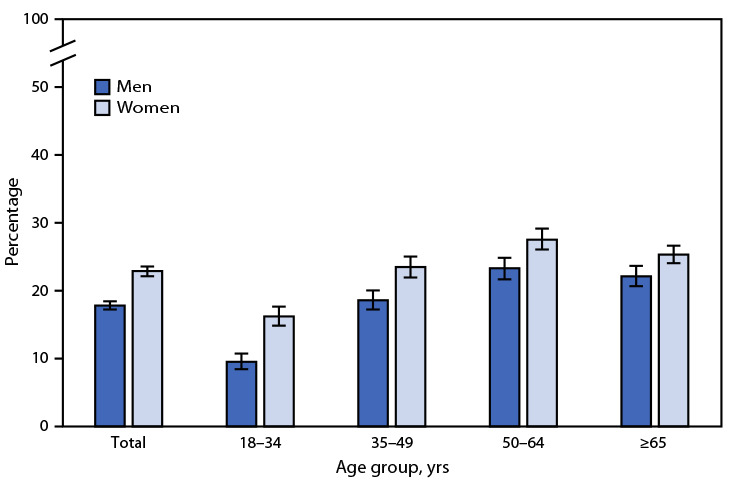
In 2022, among adults aged ≥18 years, women were more likely than men (22.9% versus 17.8%) to be advised during the past 12 months by a doctor or other health professional to increase their amount of physical activity or exercise. Percentages were higher among women than men in all age groups: 16.2% versus 9.5% among adults aged 18–34 years, 23.5% versus 18.6% among those aged 35–49 years, 27.5% versus 23.3% among those aged 50–64 years, and 25.3% versus 22.1% among those aged ≥65 years. Among both men and women, the percentage of those who were advised during the past 12 months by a doctor or other health professional to increase their amount of physical activity or exercise was lowest among those aged 18–34 years.

**Source:** National Center for Health Statistics, National Health Interview Survey, 2022. https://www.cdc.gov/nchs/nhis.htm

